# Supplementary material for: Complexome profiling of the Chlamydomonas psb28 mutant reveals TEF5 as an early PSII assembly factor
Source: Plant Cell. 2025 Mar 17;37(6):koaf055. doi: 10.1093/plcell/koaf055 (PMC12164590; doi:10.1093/plcell/koaf055)
Supplement: koaf055_Supplementary_Data [file koaf055_supplementary_data.zip › psb28_interactive.zip!/plots/intensity/qualityPlots/peptides/BCC1_Cre17.g715250.t1.2.html]

### Cre17.g715250.t1.2

Trivial name: BCC1  
SeqCoverage [median]: 11.80  
Mass: 23.58 kDa  
  
 

| Sequence | MissedCleavage | Mass [Da] | wtIntensity | muIntensity | Score |
| --- | --- | --- | --- | --- | --- |
| GIEVASPMGGTFYR | 0 | 1483.71 | 503.67 | 960.39 | 84.17 |
| LVHDTDIVEFELK | 0 | 1556.81 | 0.00 | 2785.75 | 46.48 |
|  |  |  | 503.7 | 3746.1 |  |
